# Genes Involved in Physiological Dysregulation and Decline in Resilience: Role in Alzheimer’s Disease

**DOI:** 10.1093/geroni/igab046.2228

**Published:** 2021-12-17

**Authors:** Konstantin Arbeev, Svetlana Ukraintseva, Olivia Bagley, Hongzhe Duan, Deqing Wu, Igor Akushevich, Alexander Kulminski, Anatoliy Yashin

**Affiliations:** 1 Duke University, Durham, North Carolina, United States; 2 DUKE UNIVERSITY, Durham, North Carolina, United States

## Abstract

Our recent GWAS of a composite measure of physiological dysregulation (PD) in the Long Life Family Study (LLFS) found that the top genes associated with age-related changes in PD are involved in biological pathways relevant to maintaining neural networks and brain resilience. In our prior work, PD itself was linked to resilience-related traits. Alzheimer’s disease (AD) is a heterogeneous trait and it may involve an accelerated decline in resilience with age as a contributing factor. We proposed that genes associated with aging-changes in PD and brain resilience may contribute to AD risk. We investigated interactions between SNPs in such candidate genes with AD in LLFS and Health and Retirement Study (HRS). Our analysis revealed significant interactions between SNPs in UNC5C and other genes with AD, in both LLFS and HRS. These findings support roles of genetic interactions with UNC5C gene (implemented in axon growth and neuronal apoptosis) in AD.

